# Pulmonary Sequestration Associated with Actinomycosis: A Case Report

**DOI:** 10.3390/antibiotics9100687

**Published:** 2020-10-09

**Authors:** Juan José Chaves, Fernando Polo Nieto, María Gómez-Gómez, Diana Fierro Rodríguez, Daniel García-Concha, Rafael Parra-Medina

**Affiliations:** 1 Department of Pathology, Hospital de San José, Hospital Infantil Universitario de San José, Fundación Universitaria de Ciencias de la Salud, Bogotá 110111, Colombia; jjchaves@fucsalud.edu.co (J.J.C.); jfpolo@fucsalud.edu.co (F.P.N.); mpgomez1@fucsalud.edu.co (M.G.-G.); 2 Department of Radiology, Hospital de San José, Hospital Infantil Universitario de San José, Fundación Universitaria de Ciencias de la Salud, Bogotá 110111, Colombia; dmfierro@fucsalud.edu.co (D.F.R.); dgarcia@fucsalud.edu.co (D.G.-C.); 3 Research Institute, Fundación Universitaria de Ciencias de la Salud, Bogotá 110111, Colombia

**Keywords:** pulmonary actinomycosis, bronchopulmonary sequestration, congenital malformation, pulmonary lesion

## Abstract

*Background:* Bronchopulmonary sequestration is a rare congenital malformation of the lower respiratory tract; it consists of a nonfunctioning mass of lung tissue that is irrigated by an anomalous systemic artery. The association with *Actinomyces* superinfection has not been well established. *Methods:* We present the case of a 35-year-old woman with a history of recurrent episodes of pneumonia. Based on radiological and histopathological examination, she was diagnosed with intralobar bronchopulmonary sequestration associated with *Actinomyces* infection. Promoting clinical suspicion is essential to diagnose pulmonary actinomycosis in patients with recurrent pneumonia, to improve early recognition and timely management.

## 1. Introduction

Bronchopulmonary sequestration (BPS) is a rare congenital malformation of the lower respiratory tract and represents 0.15–6.4% of congenital lung malformations [1]. It is characterized by a mass of nonfunctioning lung tissue that is supplied by an anomalous systemic artery and does not have a bronchial connection to the native tracheobronchial tree [2]. There are two forms of BPS: intralobar and extralobar; intralobar sequestration is the most common and has a reported incidence of 70–75% among all cases of sequestration [3]. Intralobar bronchopulmonary sequestrations (IBS) are incorporated into the normal surrounding lung tissue, predominantly in the posterior lateral segment of the left lung. Most cases of IBS are diagnosed at 20 years or older and there is no statistical difference in prevalence between genders [4,5]. It is known that an important factor for the recognition of this malformation is superinfection of aberrant lung tissue [6], where the association with *Actinomyces* is not clear.

Actinomycosis is a chronic endogenous and rare granulomatous infection [7]. More than 30 species of *Actinomyces* have been described. The most common causal agent in this disease is *Actinomyces israelii*, which was originally described by Kruse in 1896 as *Streptothrix israelii* [8]. The *Actinomyces* species are obligate, Gram-positive, filamentous and nonmobile anaerobic bacteria [9]. At the pulmonary level, its etiology is secondary to the aspiration of secretions from the oropharynx or gastrointestinal tract into the respiratory tract [10].

The association between BPS and *Actinomyces* superinfection has not been well studied. We report a case of BPS and pulmonary actinomycosis. 

## 2. Case Presentation

**Figure 1:**
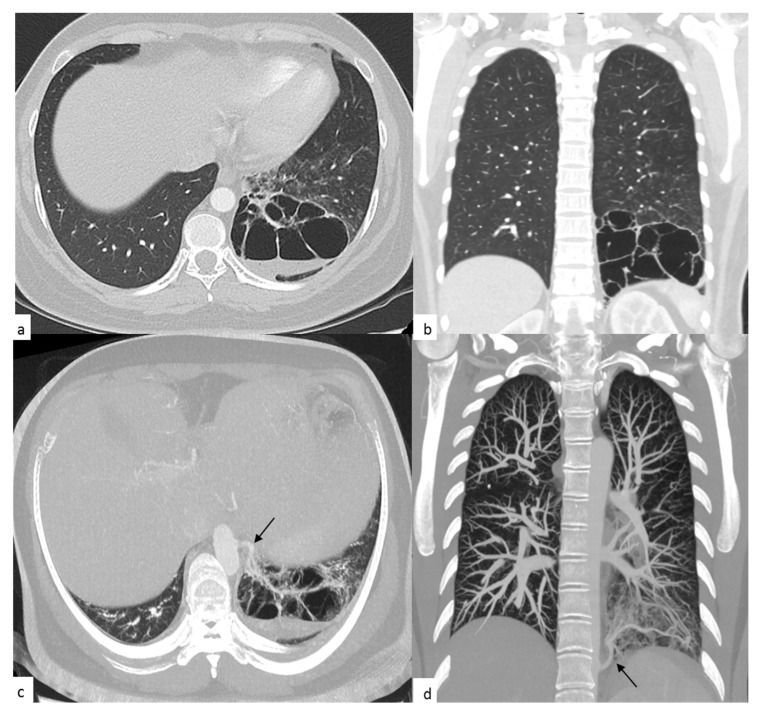
Axial (**a**,**c**) and coronal (**b**,**d**) chest computed axial tomography (CT) scan: (**a**) and (**b**): images with contrast show a hypodense, cystic avascular lesion, with multiple internal septa (multicystic) and diameters greater than 67 × 69 × 88 mm (PA × T × L); the cysts measure between 15 and 35 mm, are located in the posterior segment of the left lower lobe, and contain air-fluid levels. Peripheral parenchyma bronchial wall thickening and centrilobular micronodules are also evidenced. (**c**) and (**d**): images obtained using multiplanar reformation (MPR) outline the course of an aberrant arterial vessel (black arrow), which originates from the left lateral aspect of the thoracic aorta and supplies the left basal cystic lesion.

A 35-year-old female, who attended the outpatient clinics of the Thoracic Surgery Service, was referred by the general practitioner for presenting with dyspnea and persistent episodes of coughing. Physical examination revealed bilateral diffuse crackles upon auscultation of the lungs. She had a 4-year history of recurrent episodes of pneumonia, which had required hospitalization and antibiotic management. No information about the features of the episodes and the specific therapy she had received was available, for this was the first time she was seen in our facility and there was no access to prior clinical records. There was no past history of cigarette smoking or secondhand smoke exposure and her family history was negative. A computed axial tomography (CT) scan of the chest was requested. Three weeks later, she attended a follow-up visit, and her CT scan revealed a multicystic mass vascularized by an aberrant vessel in the posterior segment of the lower left lobe (Figure 1). The patient was scheduled to undergo a total pulmonary lobectomy of the lower left lobe, which was postponed by two months due to administrative procedures.

Upon hospital admission for surgery, blood tests revealed a high white blood cell count of 22.2 × 10^9^/L (reference range: 3.5–10.5 × 10^9^/L) with neutrophil predominance. Normal serum values were found for electrolytes, creatinine, hemoglobin, glucose levels, pH and lactic acid (Table 1).

**Figure 2:**
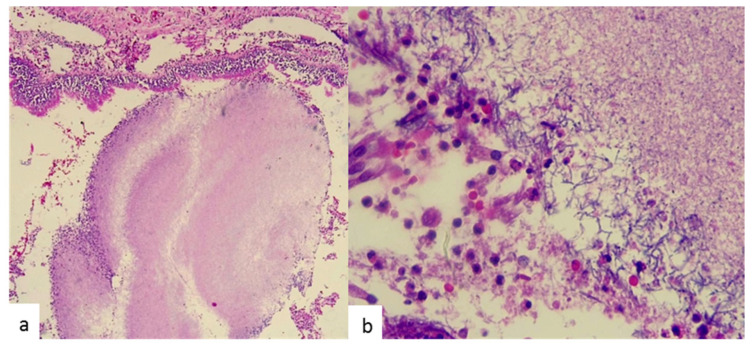
Histopathological examination of the lesion at low magnification (10×; H&E stain) shows *Actinomyces* colonies, with the characteristic “sulfur granules” (**a**), and at higher magnification (100×; H&E stain) demonstrates filamentous nonsporulating microorganisms (**b**).

A lung fragment measuring 12 × 11 × 6 cm and weighing 251 g was obtained for histopathology study. Gross examination of the resected specimen revealed irrigation by an aberrant artery to nonfunctional pulmonary tissue. Tissue sections showed two whitish multilocular cystic areas filled with purulent exudates; the largest area measured 4 × 3.5 × 1 cm and the smallest 2.5 × 2 × 1 cm. Microscopic examination by hematoxylin–eosin (H&E) staining in the tissue resected from the cystic areas evidenced multiple inflammatory infiltrates with the presence of colonies of a filamentous nonsporulating bacillus (Figure 2); Gram-positive bacteria consistent with *Actinomyces* spp. were visualized by means of the Gram staining technique using crystal violet as reagent. Other staining procedures for fungi and acid-alcohol-resistant microorganisms were negative. The lung specimen was not submitted for culture at the microbiology laboratory, for not being suitable due to prior exposure to paraffin.

One week after surgery, the patient was discharged and was given no antibiotic therapy for she was asymptomatic at that time and her white blood cell count was close to the normal reference range. A postoperative follow-up visit with the Thoracic Surgery Service to discuss basic facts about the diagnosis and for selecting and initiating a proper antibiotic regimen is pending.

## 3. Discussion

Pulmonary actinomycosis is a chronic, indolent and slowly progressive disease. The infection results from the aspiration of oropharyngeal or gastrointestinal secretions into the respiratory tract. Risk factors include poor oral hygiene, pre-existing dental disease and alcoholism [10,11], although direct and hematogenous spread are also possible [12]. Patients generally present constitutional symptoms such as fever and weight loss, and may even present recurrent episodes of pneumonia in which a germ is not isolated [13].

The association of pulmonary actinomycosis with BPS has not been well established, and only one case has been reported in the literature so far [14]; however, the identification of other microorganisms have been documented, the most prevalent being *Aspergillus*, *Mycobacterium tuberculosis*, nontuberculous mycobacteria, *Pseudomonas* and *Nocardia* [6,15,16,17,18]. Most patients with BPS and superinfection, such as in our case, are asymptomatic and have carried the congenital anomaly for years without diagnosis, being identified incidentally during a routine physical examination or by recurrent episodes of lung infection. The pathophysiological basis of BPS development is not well understood. The most accepted theory is that proposed by Pryce [19], where the fundamental trigger factor is the traction exerted by the aberrant arteries on the developing bronchial buds; however, there are hypotheses that propose an acquired origin, given its late presentation and some observations that show the development of systemic arterial collaterals in the inflammatory processes context [20].

Pulmonary actinomycosis and BPS have a nonspecific radiological presentation. The main CT findings in pulmonary actinomycosis include a mass-like consolidation, cystic lesions, cavitation and pneumonia lesions [21,22]. Histological examination and bacterial culture from a lung sample are the gold standards for diagnosis. The histopathological study demonstrates the formation of a pattern of granulomatous infection characterized by clusters of sulfur granules in 75% of cases, described in the H&E stain as basophilic masses of 0.1–1.0 mm with eosinophilic endings [23]. Microorganisms may be scarce in specimens obtained for pathological examination, so their detection requires a diligent search in multiple tissue samples. As anaerobic bacteria, *Actinomyces* need to be cultured in an anaerobic environment, where symbiotic microorganisms and contamination with other microorganisms can inhibit the growth of *Actinomyces*, so the positive rate of sputum culture is typically low [24]. Growth of *Actinomyces* is slow. The incubation period varies from 5 to 20 days. Thus, incubation of at least 10 days is required before conclusion of a negative culture [13].

Pulmonary actinomycosis is known as “the great imitator”, where the association of the clinical symptoms and radiological findings makes us consider the diagnosis of early-stage neoplastic processes and infections [25]. Within the difficulties of microscopic examination of respiratory specimens, differentiating *Nocardia* from *Actinomyces* poses a diagnostic challenge, where among some characteristics leading to the identification of *Nocardia* is the absence of granule formation and the partial staining of *Nocardia* isolates by the Ziehl–Neelsen method [26]; but cultures are the current gold standard for differentiating these microorganisms.

The management of pulmonary actinomycosis is based on the use of prolonged infusions of beta-lactam antibiotics, although surgical management may be required in some cases [27]. Even though therapy should be individualized, the standard management is high doses of intravenous penicillin for 2–6 weeks followed by oral therapy with penicillin V or amoxicillin for 6–12 months [28].

Some major limitations of our case presentation should be noted. First, as we reviewed the information contained in the medical record of our patient, we identified that several studies that could have been useful in this patient were not conducted. Second, the histopathology report was pending at the patient’s discharge, thus a proper treatment could not be initiated during hospitalization.

We present a rare case of pulmonary actinomycosis associated with BPS, which has not been well established in the medical literature. These two entities have similar clinical characteristics, such as chronicity, the presence of multiple episodes of pneumonia and nonspecific radiological presentation. Therefore, clinical diagnosis is difficult and most likely the final diagnosis is made through histopathological examination, for *Actinomyces* are difficult to culture. There is a high frequency of infection in patients with BPS, and an accurate identification of the causative organism, including *Actinomyces* spp., should be achieved.

## Figures and Tables

**Table 1:** Clinical laboratory test results.

Test	Reference Ranges in Adults	On Admission	At Discharge
Leukocytes (×10^9^/L)	3.5–10.5	22.2	10.8
Neutrophils (×10^9^/L)	1.40–6.50	20.7	7.6
Lymphocytes (×10^9^/L)	1.20–3.40	0.4	1.6
Monocytes (×10^9^/L)	0.00–1.00	1.1	1
Eosinophils (×10^9^/L)	0.00–0.70	0	0.6
Basophils (×10^9^/L)	0.00–0.20	0	0
Hemoglobin (g/dL)	12.00–17.00	12.2	9.6
Hematocrit (%)	36.00–54.00	36.6	28.4
Mean corpuscular volume (MCV) (fL)	80.00–100.00	88.5	88.9
Mean corpuscular hemoglobin (MCH) (pg)	27.00–33.00	28.7	30.1
Mean corpuscular hemoglobin concentration (MCVC) (g/dl)	32.00–36.00	32.4	33.9
Platelet count (×10^9^/L)	130–450	315	256
Urea nitrogen (mg/dL)	7–18	11	8
Creatinine (mg/dl)	0.70–1.20	0.5	0.52
Sodium (mmol/L)	137–145	142	143
Potassium (mmol/L)	3.60–5.00	3.9	3.8
Chloride (mmol/L)	98–107	106	106
pH	7.35–7.45	7.37	7.48
PO_2_ (mmHg)	90.00–100.00	155	48
PCO_2_ (mmHg)	35.00–45.00	31	33
HCO_3_ (mmol/L)	22–28	31	33
FIO_2_ (%)	-	50	21
Lactic acid (mmol/L)	0.50–1.60	0.6	0.9
Glucose, fasting (plasma) (mg/dL)	65.00–105.00	103	85
